# Drosophila: An Emerging New Approach Method (NAM) for Studying Amyotrophic Lateral Sclerosis (ALS)

**DOI:** 10.3390/cells15161432

**Published:** 2026-08-08

**Authors:** Sasha Leggett, Nitesh Sanghai, Chuying Ru, Paul C. Marcogliese, Geoffrey K. Tranmer

**Affiliations:** 1 College of Pharmacy, Rady Faculty of Health Sciences, University of Manitoba, Winnipeg, MB R3E 0T5, Canada; leggett2@myumanitoba.ca; 2 Department of Biochemistry and Medical Genetics, Rady Faculty of Health Sciences, University of Manitoba, Winnipeg, MB R3E 0T5, Canada; chuying.ru@mail.utoronto.ca (C.R.); paul.marcogliese@umanitoba.ca (P.C.M.); 3 Children’s Hospital Research Institute of Manitoba (CHRIM), University of Manitoba, Winnipeg, MB R3E 0T5, Canada

**Keywords:** *Drosophila melanogaster*, neurodegenerative diseases, new approach methodologies, amyotrophic lateral sclerosis, United States FDA

## Abstract

**Highlights:**

**What are the main findings?**

- NAMs provide ethical and efficient alternatives to vertebrate-based research.
- NAMs support the 3Rs (Replacement, Reduction, and Refinement) while advancing drug discovery.
- About 70% of human disease-related genes are conserved in *Drosophila*, supporting translational research.

**What is the implication of the main finding?**

- NAMs can accelerate therapeutic discovery while reducing animal use.
- *Drosophila* offers a scalable, cost-effective platform for ALS research aligned with regulatory trends.
- Fruit flies are widely used for proof-of-concept studies, genetic screening, and disease modeling.

**Abstract:**

*Drosophila melanogaster* (*D. melanogaster*), or fruit flies, are a commonly used model organism in the study of neurodegenerative diseases (NDs). Their short lifespan, low cost, genetic tractability, and conserved signaling and developmental pathways make them ideal for studying NDs and associated biochemical pathways. Further, flies offer the advantage of high-throughput exploratory drug and genetic screening without stringent ethical constraints. Therefore, *D. melanogaster* serves as an ideal organism for preliminary drug screening before transitioning to toxicity and efficacy studies in vertebrate models. Following the recent plan by the United States FDA (US FDA) and the National Institutes of Health (NIH) to progressively phase out preclinical drug testing in vertebrate animals and introduce New Approach Methodologies (NAMs), *D. melanogaster* has the potential to become part of the conventional drug testing pipeline in the future. This literature review focuses on the use of *D. melanogaster* models as a powerful, low-cost model organism to study superoxide dismutase 1 (SOD1)- and TAR DNA-binding protein 43 (TDP-43)-linked Amyotrophic Lateral Sclerosis (ALS), as well as previous efforts to screen drugs in SOD1- and TDP-43-expressing *Drosophila* models.

## 1. Introduction

This review examines the emerging role of *Drosophila* melanogaster as a New Approach Methodology (NAM) for ALS research, with a focus on the drug discovery pipeline. We first discuss the growing emphasis on NAMs in biomedical research and the need for improved and diversified preclinical models in ALS. We then review the features that make *Drosophila* suitable for modeling ALS pathology, with focusing on its genetic toolkit and experimentally accessible disease phenotypes. We aim to summarize current *Drosophila* models of SOD1- and TDP-43-associated ALS, highlighting their contributions to understanding disease mechanisms and evaluating therapeutic candidates through drug screening studies.

## 2. New Approach Methodologies and Drosophila

Various governmental agencies and policymakers worldwide are advocating for New Approach Methodologies (NAMs) as complementary approaches or complete replacements for animal research. While the definition for NAMs is not unified across regulatory bodies, they are generally understood to be in vitro, in chemico or in silico approaches that can be used to generate information related to chemical or pharmaceutical efficacy and safety assessment [1]. Some organizations limit NAMs to exclude any in vivo research, even in invertebrates such as nematodes and insects. However, both the U.S. FDA and National Institutes of Health (NIH) include invertebrates under examples of NAMs, with the NIH explicitly mentioning *Drosophila* as a new approach methodology.

In December 2022, the FDA Modernization Act 2.0 was signed into law. This bill essentially rescinds part of the Federal Food, Drug, and Cosmetics Act of 1938, making animal testing no longer a requirement for all drug development and approval processes [2]. This change in regulation allows for the incorporation of alternatives to animal testing, without outright banning the use of animals in drug development. The FDA Roadmap to Reducing Animal Testing in Preclinical Safety Studies cites poor prediction of drug success in patients and ethical concerns, along with financial and time-related concerns, as rationales for advocating the use of NAMs for the assessment of safety, efficacy and pharmacology rather than animal models [3]. Some key NAMs mentioned include organoids, computational modeling, AI predictive models, and ex vivo systems. The FDA aims to significantly reduce toxicity testing in animals before 2030, through encouraging the submission of NAM data, developing an open-access repository of drug toxicity data, and reducing the recommended duration of animal toxicity testing. Through this, the FDA aims for NAMs to be the primary method of preclinical safety/toxicity assessment, with the inclusion of animal models only when necessary. Following the FDA initiative, the NIH announced that it will no longer provide funding for biomedical research focused solely on animal models, and that all funding opportunities must also support human-focused approaches, such as clinical trials or NAMs [4]. Funding may also be provided for research completely excluding animal testing.

**Figure 1:**
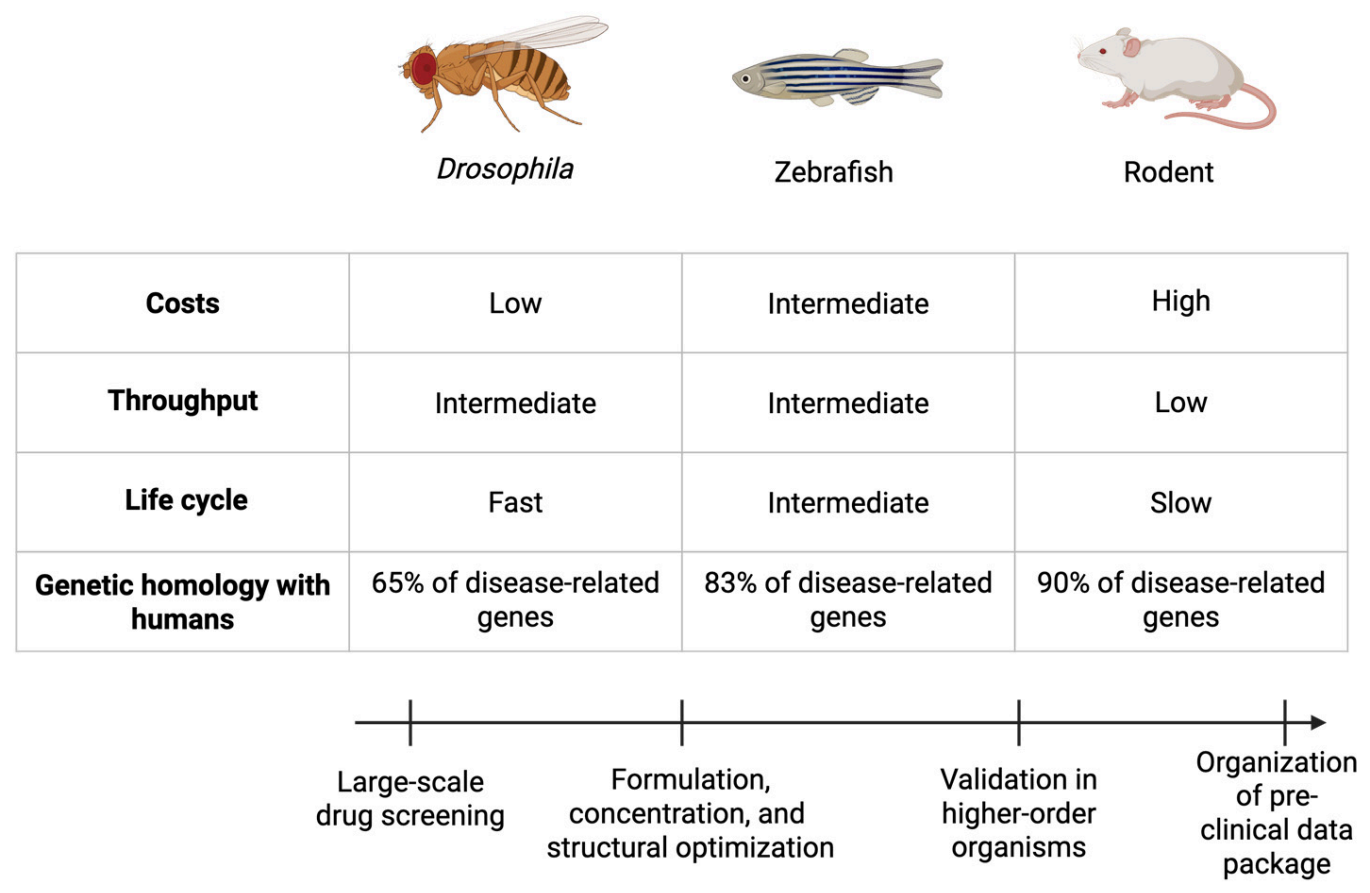
A comparison of *Drosophila,* zebrafish, and rodent models in the context of drug screening. *Drosophila* boasts low maintenance costs and superior throughput compared with rodent models, making it ideal for large-scale, rapid drug screening prior to validation in higher-order organisms. Created in BioRender. Leggett, S. (2026). Available online: https://BioRender.com/bp3hsne (accessed on 4 August 2026).

On 6 October 2025, the FDA approved the Investigational New Drug (IND) application for the experimental anticancer drug BAL0891 (SillaJen), together with the immune checkpoint inhibitor tislelizumab (BiOne Medicine) [5]. The approval is based on preclinical research using cell line studies and various AI-driven organoid models. This marks a significant milestone in the shift away from traditional animal testing and towards NAMs, including *Drosophila,* as the conventional pathway for drug development and regulatory approval. *D. melanogaster* can help researchers adhere to the 3R principles of Replacement, Reduction, and Refinement [6]. As an invertebrate model, fruit flies can partially replace higher-order mammals, or greatly reduce the amount of laboratory mice or rats needed for preclinical drug testing (Figure 1). While in-depth comparative cost analysis of *Drosophila* versus zebrafish, rodent, or other models is lacking, the FlyPower group of Brazil estimates that yearly costs associated with *Drosophila* are up to nine times and 10 times lower than cell-culture labs or mice labs, respectively [7]. Additionally, at room temperature or approximately 25 °C, a complete generation takes only about 10 to 12 days from fertilization to a sexually mature adult. Female flies are capable of laying up to 100 embryos per day [8]. In comparison, laboratory mice require an average of 12 weeks for a full generational cycle [9].

## 3. Amyotrophic Lateral Sclerosis (ALS)

Amyotrophic Lateral Sclerosis (ALS) is a fatal neurodegenerative disease (ND), causing the progressive death of both upper and lower motor neurons in the brain and spinal cord. This leads to the loss of voluntary muscle movement and speech, ultimately resulting in respiratory failure, usually within 2–5 years of diagnosis [10]. ALS is a complex, incurable disease with no single established cause. For most cases, ALS is a multifactorial disease arising from complex interactions between genetic predispositions and environmental exposures. ALS can be categorized as sporadic (sALS) or familial (fALS), with over 90% of cases being sporadic. Patients with sALS often have no clear genetic component or etiology, while most people with familial ALS acquire it through dominant genetic inheritance. Approximately 4000 Canadians suffer from ALS, and the global prevalence amounts to over 300,000 [11,12]. The average age of onset is between 58 and 63 years in sporadic ALS and 40 and 60 years in familial ALS [13]. At the molecular level, ALS has been linked to pathogenic variants in multiple genes, all of which have been implicated in cellular processes such as oxidative stress, mitochondrial dysfunction, and inflammatory mechanisms [14,15,16,17]. Additionally, exposure to toxins and military service have been proposed as environmental risk factors increasing the chance of developing ALS [18]. ALS has been linked to over 120 genes, particularly *C9ORF72* (chromosome 9 open reading frame 72), *SOD1* (superoxide dismutase 1), *TARDBP* (TAR DNA binding protein), and *FUS* (fused in sarcoma) [19]. Current primary treatments for ALS include riluzole and edaravone. Riluzole provides modest clinical benefits and is capable of extending patient survival by 2–3 months [20]. Edaravone slows the decline in ALSFRS-R (Revised ALS Functional Rating Scale) score, and some evidence suggests that edaravone may extend patient survival by over 6 months [21,22,23,24]. More recently, tofersen is an antisense oligonucleotide, recently approved for ALS through accelerated approval by the U.S. FDA in April 2023; however, its use is limited to patients with *SOD1* gene mutations, which only account for 10–20% of familial ALS cases and around 1–2% of sporadic ALS cases [17]. ALS treatment is challenging due to its multifactorial nature, rapid functional decline in patients, and the variety of pathological factors involved. In addition, intravenous injection of edaravone and intrathecal administration of tofersen can be uncomfortable and cause adverse effects, motivating the development of more stable and effective medications [25,26]. We propose that the advancement of new animal models and faster screening, such as with *D. melanogaster,* may help expedite the preclinical drug pipeline, helping expedite disease-modifying therapies to patients.

## 4. Drosophila as a Model for ALS

**Figure 2:**
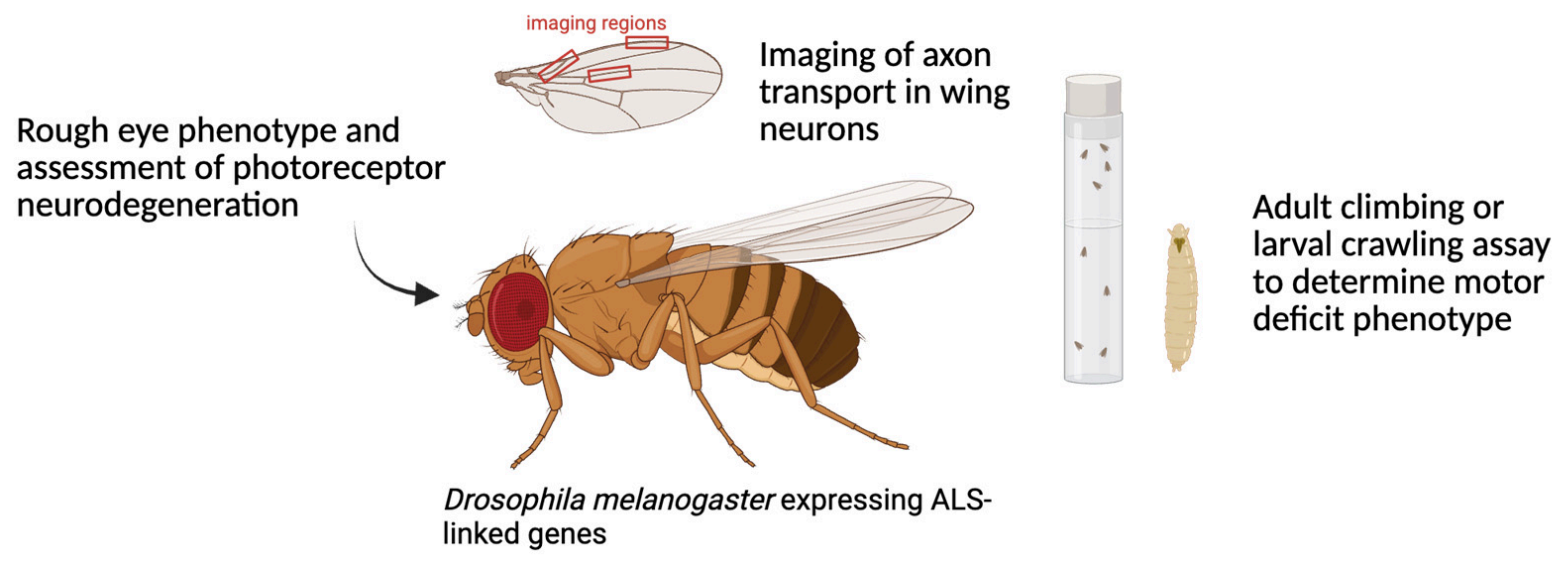
A variety of assays are used to assess the ALS phenotype in transgenic *Drosophila*. The negative geotaxis assay/climbing assay and larval crawling assay can be used to characterize reduced motor function. Wing imaging and rough eye phenotype assessment provide further insight into neuron function and ALS transgene toxicity. Created in BioRender. Leggett, S. (2026). Available online: https://BioRender.com/l1jy2t9 (accessed on 4 August 2026).

*D. melanogaster* allows for diverse genetic manipulation and straightforward observation of disease phenotypes through methods such as lifespan and climbing assays, surrogate readouts in the compound eye, and proteomics (Figure 2). Fruit flies are easy to maintain and reproduce at a rate much faster than many other animal models. Additionally, stock centers such as the Bloomington Drosophila Stock Center at Indiana University and the Harvard Transgenic RNAi project provide a comprehensive collection of various transgenic fly lines to be used in research [27,28].

Observing and imaging the *Drosophila* eye to assess disease progression is particularly useful, as approximately two-thirds of *Drosophila* genes are involved in eye development [29]. One method commonly used to determine protein toxicity in *Drosophila* is the ‘rough eye phenotype’. The *Drosophila* eye is made up of over 700 ommatidia, which are individual optical units, joined together as a hexagonal lattice to create the fly’s compound eye. Targeted expression of ALS-linked proteins in the eye can cause abnormalities in the ommatidia and overall lattice structure [30,31]. Evaluation of these abnormalities can then be used to assess gene toxicity, potentially followed by drug treatment to determine any phenotypic rescue. Rough eye phenotype screening was previously limited to qualitative evaluation, which can lead to biased results; recently, new methods have been developed for large-scale, quantitative evaluation through computer software and numerical analyses [32].

**Figure 3:**
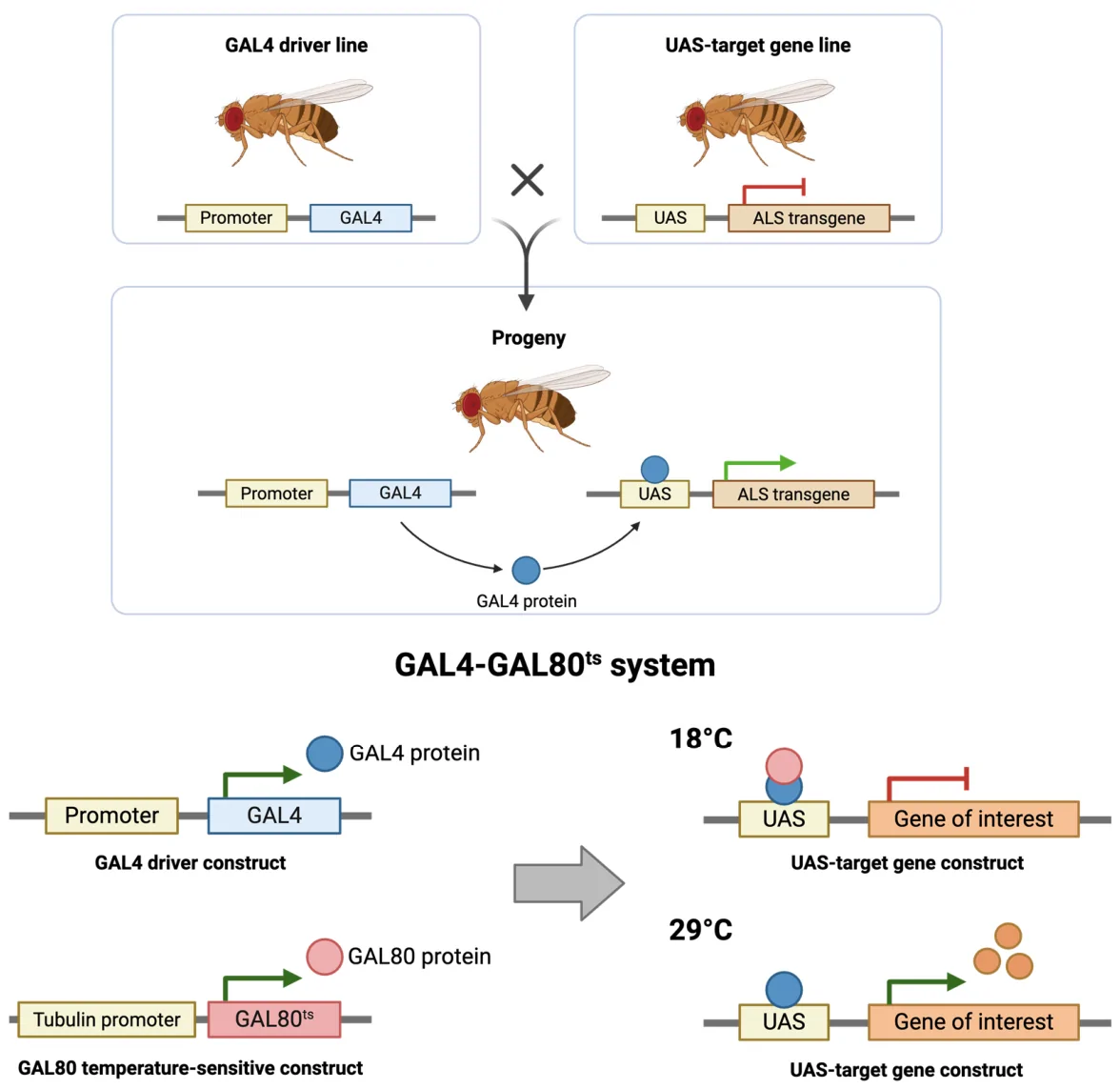
The UAS-GAL4 and GAL4-GAL80^ts^ system in *Drosophila melanogaster.* The UAS-GAL4 system is used to control spatial expression of targeted genes. In offspring, the GAL4 protein expressed in specified tissues will bind to the upstream activation sequence (UAS) and activate transcription of the ALS transgene. Created in BioRender. Leggett, S. (2026). Available online: https://BioRender.com/n25t3an (accessed on 4 August 2026).

Transgenic flies most often use the GAL4-UAS expression system (Figure 3). This system uses a driver line expressing the yeast transcriptional activator GAL4 in a specific regulatory region, along with a transgenic line carrying an upstream activation sequence (UAS)-dependent cDNA that is activated by GAL4 binding. GAL4 can be used to control the spatial and temporal expression of UAS-dependent genes. The result is fly progeny which express GAL4 in the regulatory region, thus activating the UAS-dependent transgene in a spatially controlled manner [33].

An important aspect of ALS that may sometimes be overlooked when developing animal models is the late-stage onset of symptoms. With the age of diagnosis averaging between 40 and 60 years, it may be necessary to develop models that similarly do not express ALS-linked mutated transgenes until adulthood is reached. This is possible with Temporal and Regional Gene Expression Targeting (TARGET) and the Gene Switch system. TARGET uses a tubulin promoter, leading to expression of temperature-sensitive GAL80. At lower temperatures (e.g., 18 °C), GAL80 binds GAL4, preventing activation of UAS. After induction at 29 °C, GAL80 denatures and can no longer block GAL4 from interacting with UAS, allowing transgene production. Alternatively, the Gene Switch system uses a hormone-inducible GAL4, which is inactivated until flies are fed with a nutritional medium containing RU486 (mifepristone), allowing for GAL4 activation and subsequent transgene expression [33]. With these methods of temporal control, along with the availability of different tissue-specific promoters, *Drosophila* provides various opportunities for genetic manipulation. The common GAL4 driver lines used to model ALS in *Drosophila* include D42-GAL4 for motor neuron expression, nSyb-GAL4 for Pan-neuronal, and GMR-GAL4 for the developing eye (Table 1).

Fruit flies require only 50 motor neurons to move a single leg, whereas human legs are innervated with over 150,000 motor neurons [34]. Evidently, the scale and complexity of human neuronal circuits far exceed the fruit fly. However, the simplicity of the *Drosophila* CNS, along with several neuronal and glial characteristics shared with vertebrates, make *Drosophila* an effective organism for modeling motor neuron disease.

**Figure 4:**
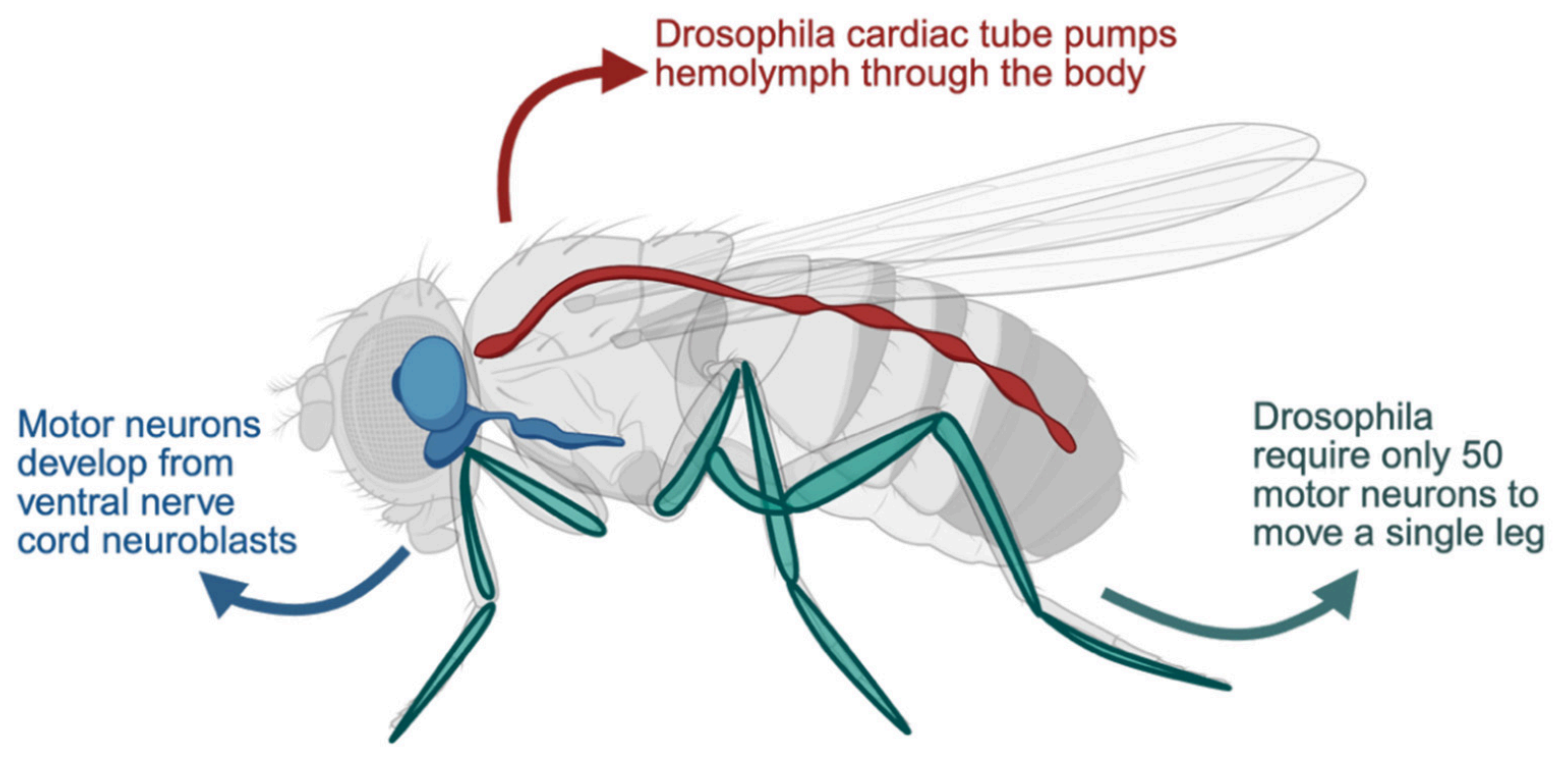
The neuroanatomy and circulatory system of *Drosophila*. Drosophila motor neuron development and circulatory system differ from those of humans in some ways; however, the simplicity and shared neuronal characteristics make *Drosophila* an effective model for motor neuron disease. Created in BioRender. Leggett, S. (2026). Available online: https://BioRender.com/huanw4w (accessed on 4 August 2026).

The circulatory system in *Drosophila* is open, and hemolymph, the equivalent of vertebrate blood, is pumped through the body cavity [35]. Surrounding the peripheral nerves are a layer of perineurial and subperineurial glia, which form a mechanical barrier and control molecular transport between circulation and the nervous tissue. This differs from the vertebrate blood–brain barrier, which is formed by brain endothelial cells together with pericytes and astrocytes as part of the neurovascular unit [36]. Nevertheless, the fly barrier conserves the physiological function of regulating exchange between the circulation and nervous system, making it a useful model for barrier function (Figure 4) [37].

*Drosophila* motor neurons develop from neuroblasts located along the ventral nerve cord, analogous to the mammalian spinal cord. In vertebrates, motor neurons are generated from progenitors found in the medio-lateral and dorso-ventral axes of the ventral neural tube [38]. In *Drosophila*, the neuronal cell bodies are unipolar and are found in the outer non-synaptic layer (cortex), displaying one primary neurite that projects into the synaptic core (neuropile). This differs from vertebrates, where the heteromultipolar neuronal cell bodies lie in the synaptic core. Additionally, humans have layers of myelin covering their ascending and descending axons. Although ALS is not a demyelinating disease, it is important to note that similar myelination is not present in *Drosophila* axons, which are instead wrapped in glia, with a different lipid composition [39,40]. A further limitation is that *Drosophila* motor neurons lack the extremely long axons characteristic of human spinal motor neurons. Since impaired axonal transport is thought to be an early pathogenic event in ALS, the fly cannot fully reproduce the demands imposed by meter-long axons [41,42]. Nevertheless, the core molecular machinery underlying axonal transport is highly conserved, making *Drosophila* a valuable model for exploring transport dysfunction while recognizing anatomical limitations.

## 5. SOD1 (Superoxide Dismutase 1)

SOD1 (Superoxide Dismutase 1) is a homodimer metalloenzyme, each monomer binding one copper and zinc ion, with dimerization occurring through hydrophobic and electrostatic interactions [43]. Each subunit of SOD1 contains four cysteine residues at amino acids 6, 57, 111, and 146, with the Cys-57 and Cys-146 residues forming a disulphide bond. Pathogenic variants in *SOD1* were the first identified cause of ALS [44]. ALS-linked *SOD1* pathogenic variants often cause either the reduction of the Cys-57/Cys-146 disulphide bond or the inhibition of metalation, leading to destabilization and monomerization rather than homodimer formation. This destabilization then leads to the formation of toxic, SOD1-containing aggregates [45,46]. SOD1 functions as an antioxidant enzyme, protecting against reactive oxygen species (ROS) in the cell by catalyzing the conversion of superoxide into oxygen and hydrogen peroxide, as defined in Equation (1). ROS are known to oxidize various biomolecules, such as DNA and proteins, thus preventing the proper function of enzymes or inducing mutations in DNA. Furthermore, ROS have the potential to cause necrosis or apoptosis [47]. Pathogenic variants in *SOD1* cause targeted pathology due to the abundance of SOD1 in various neurons, particularly in the axons and dendrites of motor neurons [48]. To date, more than 200 ALS-associated *SOD1* variants have been identified [49,50]. This makes it the most common, and not surprisingly, the most comprehensively studied gene found in fALS [44]. *SOD1* variant pathology may be due to loss-of-function, gain-of-function, or a combination of mechanisms. Surpassing the loss of antioxidant function, aberrant oligomerization of SOD1 protein has been shown to cause a gain of pro-oxidant activity following cleavage of intermolecular disulfide bonds [51,52]. The severity of disease attributed to different *SOD1* variants varies by age of onset and survival time after diagnosis [53,54]. *SOD1* variants can cause direct misfolding of the protein. Alternatively, post-translational modifications that disrupt quaternary structure or induce oxidation can also cause misfolding, leading wild-type SOD1 to acquire similar toxicity seen in pathogenic SOD1 [55].

(1) $${Cu}^{2+},\;{Zn}^{2+}-SOD1+O_{2}^{-}↔{Cu}^{+},{Zn}^{2+}-SOD1+O_{2}$$

$${Cu}^{+},{Zn}^{2+}-SOD1+O_{2}^{-}+2H^{+}↔{Cu}^{2+},{Zn}^{2+}-SOD1+H_{2}O_{2}$$

In illustrating the biological chemical reaction above, this shows the SOD1-catalyzed disproportionation reaction of superoxide to oxygen and hydrogen peroxide. The Ala4Val (A4V) SOD1 variant is the most common *SOD1* pathogenic variant in North America and one of the most clinically severe variants associated with dominant inheritance and reduced survival in ALS. It is characterized by extreme protein instability and aggregation, causing rapid disease progression [56]. According to a 2016 study by Bali et al., ALS patients with SOD1 non-A4V variants demonstrated a mean survival time of 6.8 years, compared to only 1.2 years for patients with SOD1 A4V variants. None of the 35 study participants harboring SOD1 A4V survived four years after diagnosis [57]. The SOD1 A4V is significantly less stable than wild-type SOD1, causing both variant homodimers and heterodimers of wild-type/variant monomers, which lead to neurotoxic aggregates [58]. These aggregates are unable to respond efficiently to oxidative stress (loss-of-function), leading to increased oxidative damage, mitochondrial dysfunction, and eventual motor neuron death [59,60]. Based on research done by Perri et al., the formation of these aggregates is only a part of the cellular pathology in SOD1 A4V-linked ALS [61]. The team showed that SOD1 A4V requires intact residues at Cys-6 and Cys-111 to form inclusions and to induce endoplasmic reticulum (ER) stress, which activates the unfolded protein response and can induce apoptosis. Research conducted by Atkin et al. shows that SOD1 A4V causes ER stress and inhibition of ER-Golgi protein transport before the formation of aggregates [62]. Both of these studies provide insight into how inclusion formation and ER stress are not directly related but are induced by the SOD1 A4V protein in ALS.

The Gly85Arg (G85R) SOD1 variant has also been shown to cause dominantly inherited ALS with rapid clinical progression. Bruijn et al. demonstrated, using a transgenic mouse model, that while the mutated SOD1 G85R proteins accumulate in the spinal cords of affected mice, the mutation has no significant effect on overall SOD1 activity. Similarly to SOD1 A4V, the G85R causes SOD1-containing aggregates in mouse motor neurons and astrocytes [63]. These SOD1 G85R-positive inclusions are likely related to the decreased affinity for zinc in the mutated protein, causing destabilization and pathogenic aggregation through misfolding. In a squid giant synapse model, human SOD1 G85R was also shown to cause aberrant calcium signaling, causing synaptic dysfunction [64]. Chelation of presynaptic calcium restored synaptic function, providing evidence that the mutation may cause a toxic gain-of-function mechanism related to presynaptic calcium accumulation. A brief description of the pathological consequences and clinical outcomes of the A4V and G85R variants, along with three other representative variants, is included in Table 2.

## 6. SOD1 ALS Models in Drosophila

In 2025, Liguori and team developed two *Drosophila* models expressing either hSOD1 A4V or hSOD1 G85R pan-neuronally (elav-GAL4) [72]. Both variants led to reduced survival and motor performance, likely through increased oxidative stress. Interestingly, the researchers analyzed the metaphase chromosomes of third-instar larvae and found that chromosome aberrations increased from 0.40% to 1.89% and 2.28% in hSOD1 A4V and hSOD1 G85R flies, respectively. The researchers noted that if this trend of chromosomal aberration can be validated in higher organisms or patients, it could be of use in early diagnosis and treatment. Similar models expressing the same two variants in motor neurons (D42-GAL4) were developed by Watson and coworkers, and again showed reduced climbing ability [73]. Of note, this loss of climbing activity was not correlated with the loss of motor neurons and instead seemed to be linked to progressively abnormal synaptic transmission. Additionally, nearby glia that did not express SOD1 themselves showed upregulation of Hsp70 and Repo, indicating a stress response.

In 2016, Gallart-Palau and team developed a transgenic fly model expressing hSOD1 G93A protein in thoracic muscles using the 24B-GAL4 driver line [74]. The group determined that the 24B-GAL4>UAS-hSOD1 G93A flies exhibited misfolded isoforms of hSOD1 protein within the thoracic fibers, which in turn caused mitochondrial disorganization and dysfunction. This led to impaired climbing ability and a shortened lifespan. Additionally, 30–40% of the generated 24B-GAL4>UAS-hSOD1 G93A flies displayed a malformed wing phenotype, causing an inability to fly or jump. Similarly, expression of *Drosophila* SOD1 G85R in multidendritic (MD) neurons of the peripheral nervous system led to reduced mitochondria in MD sensory axons, along with lower mitochondrial content and morphological defects at synapses. This culminated in compromised mitochondrial function, demonstrated by increased oxidative stress in neurons [75]. The researchers stressed the importance of conducting further studies with refined drivers to develop a more detailed understanding of the mitochondrial pathology involved, along with the potential for the discovery of novel therapeutics with the ability to prevent or reverse mitochondrial dysfunction.

Huang and team created *Drosophila* models with *tau* and hSOD1 WT or hSOD1 A4V co-expressed either pan-neuronally (elav-GAL4) or in the developing eye (GMR-GAL4) [76]. Co-expression of *tau* and hSOD1 WT or hSOD1 A4V caused enhanced degeneration compared to tau alone, which was observed through reduced lifespan, mobility, and an increased number of vacuoles in the brain. The mechanism through which hSOD1 WT or hSOD1 A4V causes enhanced tau-related neurodegeneration is likely hyperphosphorylation, confirmed by both the elevated tau phosphorylation levels as well as hSOD1 WT and hSOD1 A4V’s inability to potentiate neurodegenerative effects in hypophosphorylated tau (S2A) protein. Generally, the effects of the A4V mutation were more severe than those of wild-type SOD1, indicating that alterations in tau metabolism in ALS patients may be related to the effects of SOD1 A4V. Although the clinical relevance of tau dysregulation in ALS remains uncertain, this study illustrates how Drosophila modeling can be used to identify previously unrecognized interactions between ALS-associated proteins and other neurodegeneration-related pathways.

The advantages of modeling ALS in *Drosophila* can then be examined through the screening of potential therapeutic candidates and the assessment of mitochondrial abnormalities, mortality, motor deficits, eye degeneration, and other phenotypic rescue. Multiple studies have evaluated the neuroprotective effects of different antioxidant molecules in *Drosophila* models with familial ALS-linked human SOD1 A4V and G85R variants. Many early proof-of-concept studies used antioxidant compounds because oxidative stress is a well-established component of ALS pathology [16]. Although antioxidant therapies have generally failed to demonstrate substantial clinical benefit in ALS patients [77], these studies were important for validating *Drosophila* models as platforms capable of detecting pharmacological rescue of disease-associated phenotypes and for investigating mechanisms of oxidative stress in ALS. Both the antioxidants α-lipoic acid and fisetin were shown to extend lifespan, improve motor function, and activate the ERK pathway in D42-GAL4>UAS-hSOD1 G85R fly models [78,79]. Furthermore, flies treated with fisetin had fewer SOD1 aggregates in their brain tissue than untreated flies. Based on former findings demonstrating that fisetin is capable of reducing tau protein via autophagy activation in an Alzheimer’s disease model, the researchers propose that fisetin may scavenge human SOD1 by similar mechanisms. Other antioxidants, such as withania somnifera, γ-oryzanol, and urate, all similarly rescued motor behavior and lifespan in D42-GAL4>hSOD1 G85R flies [80,81,82].

Recently, Liguori and team identified the approved antidepressant clomipramine as a potential drug-repurposing candidate in elav-GAL4>hSOD1 A4V or G85R fly models [83]. Treatment with 0.5 mM clomipramine in fly nutritional medium improved survival, attenuated DNA damage, and reduced inflammatory markers in both models. The relationship between DNA damage and ALS pathogenesis remains incompletely understood. However, the observed DNA lesions may reflect downstream cellular stress associated with mutant SOD1 expression. DNA damage in ALS has been linked to oxidative stress, protein aggregation, and impaired cellular homeostasis [84]. Interestingly, administration of clomipramine improved motor performance in SOD1 G85R flies but reduced motor performance in SOD1 A4V flies, highlighting potential variant-specific differences in the drug response of ALS SOD1 models. The study found that the worsened motor performance in SOD1 A4V flies correlated with a reduction in acetylcholinesterase (AChE) activity and noted that antidepressants such as clomipramine may inhibit AChE and subsequently induce cholinergic crisis, causing muscle weakness and paralysis [85,86]. Mianserin at the same dose ameliorated lifespan in both mutant models but showed no other significant improvements. Structures of each compound, along with a summary of their therapeutic properties when tested in *SOD1* fly models, are presented in Table 3.

## 7. TDP-43

TDP-43, or Transactive response DNA binding protein 43 kDa, was originally discovered as a modulator of HIV-1 gene expression, but has since been implicated in ALS pathology [88]. Encoded by *TARDBP*, TDP-43 is one of the major protein components found in pathological ALS inclusions, seen in up to 97% of sporadic ALS patients [89]. TDP-43 is a structurally classic RNA-binding protein, consisting of an N-terminal domain (NTD) and two RNA recognition motifs (RRM1 and RRM2), along with a C-terminal domain. TDP-43 also contains a nuclear localization sequence, which promotes TDP-43 localization to the nucleus and connects the RRM1 domain with the N-terminal domain [90]. Although the exact mechanism behind TDP-43 proteinopathy in ALS remains unclear, it has been shown that the proteinopathy leads to two connected consequences. In a loss-of-function mechanism, the relocalization of TDP-43 from the nucleus to the cytoplasm causes transcriptional dysregulation and loss of TDP-43’s innate autoregulatory functions, leading to the overexpression of TDP-43. Excess TDP-43 in the cytoplasm forms aggregates and stress granules of ubiquinated and hyperphosphorylated TDP-43, again leading to loss-of-function of TDP-43 [91,92]. When TDP-43 is depleted from the nucleus upon overexpression, a neurodegenerative phenotype is observable in a *Drosophila* ALS-TDP model even before the presence of TDP-43 inclusion bodies [92,93,94]. *TBPH* is the *Drosophila* homolog of human TDP-43. Although there exist differences in the primary protein sequences of the two genes, TBPH and TDP-43 demonstrate significant similarity in terms of function. TBPH and TDP-43 both preferentially bind UG-repeat sequences and have overlapping splicing inhibitory effects, which are dependent on the C-terminal region [95]. These factors make *Drosophila* a promising candidate for the modeling of TDP-43-linked ALS in animals.

## 8. TDP-43 ALS Models in Drosophila

There are several approaches to developing a TDP-43-linked ALS *Drosophila* model. Each of these methods should be interpreted according to the specific aspect of TDP-43 pathology they reproduce. In ALS, TDP-43 proteinopathy is characterized primarily by nuclear depletion, cytoplasmic mislocalization, and aggregation of TDP-43. Consequently, models involving overexpression or mislocalization leading to cytoplasmic aggregate formation of wild-type or mutant human TDP-43 may more directly reproduce key pathological features. Disruption or complete knockout of the *Drosophila* TDP-43 homolog, TBPH, provides a complementary loss-of-function approach. One method involves disrupting or completely knocking out the *TBPH* gene. Homozygous deletions in *TBPH*, which create null alleles, caused over 30% of fruit flies to be unable to develop past the pupal stage, with the surviving flies showing impaired locomotive behavior and a shorter lifespan [96]. Supporting the idea that *Drosophila* TBPH and human TDP-43 have overlapping functions, overexpression of hTDP-43 rescued the TBPH deficiency phenotype [97]. Other studies have also confirmed that homozygous null alleles for *TBPH* display either a semi-lethal or fully lethal phenotype [31,98]. The presence of a TBPH deficiency phenotype, along with phenotypic rescue after reintroducing TBPH or hTDP-43, suggests a loss-of-function component in TDP-43 pathology in ALS. However, complete TBPH loss does not directly model ALS because TDP-43 is not completely absent in affected neurons. Instead, TBPH-null models are useful for defining the physiological functions of TDP-43 and investigating the consequences of reduced TDP-43 activity.

Conversely, *Drosophila* models which overexpress TDP-43 in specific regions can be developed using the GAL4-UAS system. Directed overexpression of TBPH/TDP-43 in the *Drosophila* eye using GMR-GAL4 has been shown to cause similar eye defects. Specifically, both TBPH and hTDP-43 overexpression cause retinal toxicity and protein aggregates in the axons of the eye [31,93,97,98,99]. Localization of TDP-43 expression to the eye allows for a simple visual assessment of toxicity without concerns about fly viability. Similarly, TDP-43 overexpression or RNAi-mediated TBPH knockdown can be directed to the mushroom body (MB) neurons using OK107-GAL4. The MB functions as the primary learning and memory center in *Drosophila* [100] Overexpression of TBPH/hTDP-43 in MB neurons causes learning deficiency along with axonal and neuronal loss. RNAi-mediated TBPH knockdown in *Drosophila* does not affect structure, but it does cause learning deficiency [99,101]. Overexpression of wild-type or variant hTDP-43 in motor neurons causes impaired viability, progressive motor deficits, formation of cytoplasm-localized aggregates, and axon swelling [31,93,98,101,102,103]. Cytoplasmic and nuclear accumulations of TDP-43 were both toxic to neurons, with the neurodegenerative phenotype observable even before the presence of detectable inclusions [93,97]. Even without a visible rough eye phenotype in GMR-GAL4>TDP-43 flies, the underlying pathology can be observed through electron microscopy, phototaxis assay, and histological techniques. Although Hanson and team were unable to see a visible eye phenotype in their flies expressing hTDP-43 at a moderate level with GMR-GAL4, transmission electron microscopy revealed severe rhabdomere disruption [31]. Similarly, Miguel and coworkers were able to determine that GMR-GAL4>TDP-43 flies had disruptions in the external lattice of the eye, along with the loss of some interommatidial bristles [97]. Other studies have also used retinal sections stained with toluidine blue as evidence of retinal cell loss, regardless of surface phenotype [93,99]. Interestingly, after conducting a phototaxis assay on their flies overexpressing TBPH in the eye, Cragnaz and team found that these flies were not attracted to light, indicating that they are likely blind [104]. A phototaxis assay is conducted by placing flies into the stem of a Y-maze, with one arm of the Y exposed to light, and the other remaining dark. Results are determined by the percentage of flies which move towards the light arm. To ensure that the reduced movement towards light was unrelated to any underlying motor impairment, the researchers also conducted climbing assays and determined that all the cohorts tested were of similar climbing ability to their wild-type control. Together, these experiments show TDP-43’s toxic effect on the *Drosophila* eye. Additionally, they show the range of methods available for the validation of neurodegenerative proteinopathy in *Drosophila*. It is noteworthy to mention here that the blindness phenotype is used as a readout of neurotoxicity and neuronal degeneration rather than as a representation of a specific ALS symptom.

TDP-43-based *Drosophila* models enable the screening of various compounds to rescue ALS-like pathology. In 2021, Cragnaz et al. repurposed various drugs in an aggregation-prone TDP-43 model causing the sequestration of protein and the formation of TDP-positive aggregates [105]. The most promising candidate was the antipsychotic, thioridazine, that promoted aggregate clearance and a significant improvement in climbing ability in adult flies. In 2023, Piccolo and team screened ten drugs for repurposing in ALS using a TDP-43 *Drosophila* model and identified low-dose fingolimod as the most promising candidate [106]. Fingolimod is an oral immunomodulator used for the treatment of multiple sclerosis [107]. They found that 1 µM fingolimod treatment ameliorated larval motor deficit through reduction in TDP-43 levels in a wild-type TDP-43 overexpression model. The beneficial effects of fingolimod treatment were less substantial in a TDP-43 G348C model, potentially due to the G348C variant’s resistance to degradation. Of note, treatment with fingolimod did not lead to significant improvement in the number of boutons in neuromuscular junctions (NMJs) of ALS-TDP flies, suggesting that the drug’s effects on locomotive ability are independent of NMJ morphology. The researchers also included riluzole and edaravone, two of the three currently approved ALS treatments, in their drug screening platform. Edaravone at 1 µM, but not 10 µM, increased the eclosion rate of TDP-43 G348C transgenic flies and the crawling distance of TDP-43 WT larvae but did not show improvement in all fly types compared to fingolimod. Riluzole had no significant effects on eclosion or crawling rate at either dose tested. The limited and phenotype-dependent effects of riluzole and edaravone in these fly models should be considered when evaluating its translational potential. These finding do not necessarily indicate that the model lacks translational relevance. Both drugs provide modest clinical benefits in ALS and may not be expected to produce robust reversal of disease-associated phenotypes in a single experimental model. The absence of a measurable effect may reflect limitations related to drug exposure, dose, assay sensitivity, or the specific disease mechanisms in TDP-43 ALS. The variable responses observed across TDP-43 genotypes and phenotypic endpoints may also provide useful information regarding disease heterogeneity and the context-dependent activity of candidate therapeutics. These models are therefore best viewed as tools for mechanistic discovery and early-stage therapeutic prioritization rather than as stand-alone predictors of clinical efficacy.

In 2015, Joardar and team repurposed the PPARγ agonist pioglitazone, demonstrating its neuroprotective ability in an ALS-TDP *Drosophila* model [108]. Although 1 µM pioglitazone was unable to improve locomotor dysfunction in the SOD1 model, it was capable of partially mitigating toxicity in glia and motor neurons upon glial (repo-GAL4) or motor neuronal (D42-GAL4) overexpression of TDP-43. To further determine the mechanism by which pioglitazone protects against TDP-43-related neurodegeneration, the researchers compared the metabolic profiles of flies overexpressing TDP-43 in motor neurons raised on drug food versus DMSO, along with those of wild-type flies. The only metabolite significantly restored by pioglitazone was N-acetylglutamine. N-acetylglutamine is an acetylated, stabilized analog of glutamine, and, based on a 2022 study, is an increased metabolite in both TDP-43 WT and TDP-43 G298S *Drosophila* models [109]. In a cohort of almost 400 patients with sporadic ALS, 40% were found to have increased levels of glutamate in their cerebrospinal fluid, supporting the hypothesis of glutamate excitotoxicity as a mechanism of motor neuron degeneration in ALS [110]. These findings suggest a possible involvement of altered glutamate metabolism between *Drosophila* models and ALS patients, along with reduced N-acetylglutamine levels after treatment and improvement of locomotor deficit in flies, which further support the efficacy and practicality of fruit fly ALS modeling. Another case of drug repurposing in an ALS-TBPH *Drosophila* model is the immunosuppressant rapamycin [111]. Rapamycin showed partial prevention of ALS pathology, with an increase in average lifespan from 23 days to 30+ days at a concentration of 200 µM. Moreover, administration of 400 µM rapamycin improved motor deficits and was able to reduce the number of neurons displaying TBPH-positive aggregates. Although rapamycin did not increase the neuron counts in flies overexpressing TBPH in motor neurons, its therapeutic effects were linked to the activation of autophagy [112].

In 2019, François-Moutal and team used in silico docking of the TDP-43 RNA-protein interface to show that its first RNA recognition motif (RRM1) is a druggable site [113]. Using virtual screening of fifty thousand compounds, the group then determined that rTRD01 is a small molecule capable of reducing locomotor deficits, improving larval turning time in the TDP-43 G298S transgenic model expressing the protein in motor neurons. Further work from the same group using the same virtual screening method showed that nTRD22, a small molecule able to target the N-terminal domain of TDP-43, causing allosteric modulation of TDP-43’s RNA-binding domain, leads to decreased RNA binding and mitigated locomotor deficit in a D42-GAL4>TDP43 G298S *Drosophila* ALS-TDP43 model [114].

In 2024, Droppelmann et al. demonstrated that an N-terminal fragment of rho guanine nucleotide exchange factor (RGNEF), NF242, interacts with TDP-43 and partially mitigates the neurodegenerative phenotype in a TDP-43 overexpression ALS model of *Drosophila* [115]. This includes the improvement of motor defects and an increase in lifespan. The researchers noted that NF242 and wild-type TDP-43 tended to co-aggregate in both the nucleus and cytosol of neurons and suggested that NF242 has a higher affinity for pathological TDP-43 over soluble TDP-43. This is supported by the observation that high amounts of NF242 were necessary to inhibit regular RNA binding to the soluble TDP-43. The researchers proposed that NF242, through binding pathological TDP-43, prevents TDP-43 from sequestering other proteins or RNA and inducing toxicity. The potential to achieve a therapeutic effect not by eliminating aggregates but by minimizing their toxic gain-of-function may provide an effective way to delay or modify ALS pathogenesis. A summary of the therapeutic compounds tested in *Drosophila* models of ALS-TDP is shown in Table 4.

## 9. Conclusions and Perspectives

Studies of SOD1 and TDP-43 *Drosophila* ALS models confirm the toxicity of both proteins and reiterate previous findings in murine models and patients. ALS is an incurable disease lacking efficient treatment; however, large-scale drug screenings in *Drosophila* may provide a path towards novel therapeutic molecules as effective as a NAM. *Drosophila melanogaster* may be particularly useful in the hypothesis-testing phase of drug discovery and in exploring the molecular mechanisms of active compounds. This can accelerate preclinical drug optimization and efficacy testing while lowering costs and minimizing ethical concerns associated with vertebrate animal testing. The studies reviewed here demonstrate that *Drosophila melanogaster* can reproduce several conserved molecular and functional features relevant to ALS, including mutant SOD1 and TDP-43 toxicity, protein aggregation, oxidative stress, progressive motor impairment, and reduced survival. The extensive genetic toolkit available in flies enables disease-associated variants to be expressed or manipulated in defined cell populations and at specific developmental stages. These capabilities permit rapid investigation of pathogenic mechanisms and the identification of genetic or pharmacological modifiers within an intact organism.

The principle value of *Drosophila* as a New Approach Methodology (NAM) is therefore not that it fully reproduces human ALS or replaces vertebrate models, but that it provides a rapid, genetically tractable, and comparatively inexpensive in vivo system for hypothesis testing and early-stage therapeutic discovery. In contrast to cell-based models, flies retain interactions among neurons, glia, muscle, and other tissues and allow for candidate interventions to be evaluated simultaneously across multiple organism-level outcomes, including locomotor function and survival. The short generation time, availability of large genetic resources, and relatively low experimental cost also facilitate testing across multiple disease-associated variants, genetic backgrounds, doses, and candidate compounds. These features make flies particularly well suited to identifying and prioritizing mechanisms and therapeutic candidates before more resource-intensive validation.

Rodent models remain essential for investigating aspects of ALS that cannot be adequately represented in *Drosophila*. Mammalian models provide greater anatomical and physiological similarity to humans and are better suited to studying long-projecting motor axons, corticospinal and upper motor neuron involvement, neuron-glia and immune interactions, pharmacokinetics, and treatment delivery. These differences are particularly important because axonal transport failure and interactions among motor neurons, astrocytes, and microglia contribute to ALS pathogenesis. Rather than a replacement, the comparative strengths of *Drosophila* and rodent models support complementary, tiered approaches to ALS research. Flies can be used to rapidly generate and compare disease-associated variants, identify genetic modifiers, test mechanistic hypothesis, and prioritize compounds according to their ability to improve disease-relevant phenotypes in vivo. The most promising findings can then be evaluated in mammalian systems. Such a strategy may reduce the number of vertebrate experiments required, improve the selection of candidates entering costly rodent studies, and increases the efficiency of preclinical drug development.

*Drosophila* is more than a model organism; it is a practical and scientifically robust NAM for the future of ALS research. Harnessing its genetic conservation, efficiency, and ethical advantages will help drive a new era of translational discovery with reduced dependence on vertebrate models.

## Figures and Tables

**Table 1:** Various GAL4 driver lines typically coupled with mutant UAS-SOD1 responder lines to create SOD1-linked ALS models in *Drosophila*. The same driver lines are also often used for TDP-43-linked ALS models. All information on expression patterns was collected through FlyBase (FB2025_05).

D42-GAL4	Motor Neuron Expression
OK371-GAL4	
OK6-GAL4	
C164-GAL4	
nSyb-GAL4	Pan-neuronal expression
elav-GAL4	
GMR-GAL4	Developing eye expression
repo-GAL4	Pan-glial expression
24B-GAL4	Thoracic muscle fiber expression
MD-GAL4	Multidendritic (MD) neuron expression
MHC-GAL4	Muscle, indirect flight muscle cell expression

**Table 2:** Representative common and extensively studies SOD1 variants associated with ALS. Pathogenic SOD1 variants can differentially affect protein folding, conformational stability, and aggregation propensity, contributing to variation in downstream cellular dysfunction and clinical phenotype. This table summarizes the reported molecular effects, proposed pathogenic consequences, and clinical characteristics of five representative variants, chosen by their clinical or experimental relevance.

SOD1 Variant	Effects on SOD1 Protein	Clinical Outcome	References
A4V (p.Ala5Val)	Destabilizes SOD1 dimer, increases misfolding and aggregation propensity. Enzymatic activity may remain relatively preserved.	Typically associated with rapidly progressive ALS with short survival, particularly common in North American populations.	Rosen et al., 1993 [44] Cudkowicz et al., 1997 [65] Bali et al., 2017 [57]
D90A (p.Asp91Ala)	Subtle structural changes reducing conformational stability and promoting misfolding.	Often associated with slowly progressive, predominantly lower-motor-neuron disease.	Andersen et al., 1995 [66] Li et al., 2016 [67]
I113T (p.Ile114Thr)	Destabilizes SOD1 protein, increases misfolding and aggregation propensity. Maintains substantial enzymatic activity.	Variable age at onset and disease duration, with substantial intra- and interfamilial variability.	Cudkowicz et al., 1997 [65] Prudencio et al., 2009 [68] Opie-Martin et al., 2022 [69]
G85R (p.Gly86Arg)	Disrupts SOD1 folding and stability and reduces zinc binding. Produces immature, metal-deficient protein leading to aggregation.	Generally associated with rapid progression when expressed in mice, although clinical presentation and disease duration can vary.	Rosen et al., 1993 [44] Wang et al., 2002 [70] Prudencio et al., 2009 [68]
G93A (p.Gly94Ala)	Subtle structural changes while maintaining substantial enzymatic activity, but with increased conformational instability and aggregation.	Clinical phenotype is variable and G93A is a relatively uncommon variant. However, it is used extensively in experimental models.	Gurney et al., 1994 [71] Prudencio et al., 2009 [68] Opie-Martin et al., 2022 [69]

**Table 3:** Summary of different compounds tested in SOD1 mutant fly models and their therapeutic effects.

Compound	Structure/Description	Observations and Results
α-lipoic acid	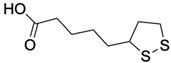	Antioxidant and neuroprotective effects demonstrated through improvement of lifespan and motor activity in the D42-GAL4>hSOD1 G85R fly model [78].
Fisetin	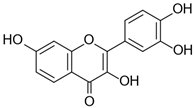	Antioxidant and neuroprotective effects demonstrated through improved survival, motor activity, and antioxidant enzyme activities in a D42-GAL4>hSOD1 G85R fly model [79].
γ-oryzanol	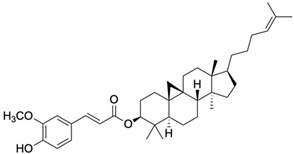	Antioxidant and neuroprotective effects demonstrated through improved survival, motor activity, and reduced oxidative damage in a D42-GAL4>hSOD1 G85R fly model [82].
Urate	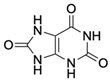	Antioxidant and neuroprotective effects were demonstrated through improved survival, motor activity, and reduced oxidative damage in a D42-GAL4>hSOD1 G85R fly model [81].
Withania somnifera	Also known as Ashwaganda, a traditional herb with bioactivity and potential anti-inflammatory properties. The plant contains various steroidal lactones, called withanolides, which are used in alternative medicine [87].	Enhancement of survival rate, motor ability, and rescue of abnormal mitochondrial morphology in a D42-GAL4>hSOD1 WT fly model [80].
Clomipramine	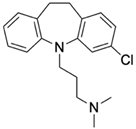	Improvement of lifespan in elav-GAL4>hSOD1 G85R and elav-GAL4>hSOD1 A4V fly models. Motor deficit improvement only in G85R flies. Reduction in inflammation markers and attenuation of DNA damage [83].
Mianserin	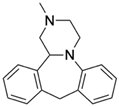	Significant (22% and 13%) lifespan improvement in elav-GAL4>hSOD1 A4V and elav-GAL4>hSOD1 G85R flies respectively. No other significant benefits were observed, and an increase in inflammatory markers was observed [83].

**Table 4:** Summary of different compounds tested in SOD1 mutant fly models and their therapeutic effects. A superscript of TS indicates that the driver is temperature sensitive, allowing for controlled induction of gene expression through temperature control.

Compound	Structure/Description	Observations and Results
Thioridazine	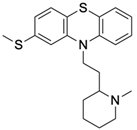	At 50 µM during larval stage and 0.4 mM in the adult stage, treatment caused significant improvement of locomotor deficit in an elav-GAL4>FLAG-TDPF4L-12XQ/N (aggregation-causing TDP-43 mutation) fly model [105].
Fingolimod	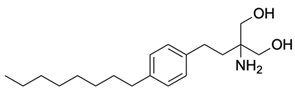	At a 1 µM concentration, improved pupal lethality and locomotor deficit in D42-GAL4>TDP-43 WT and D42-GAL4>TDP-43 G348C flies. No significant improvement in NMJ morphology. Reduction in level of TDP-43 WT with an elav-GS driver [106].
Pioglitazone	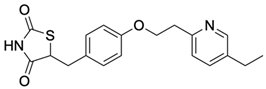	At a 1 µM concentration, improved larval locomotor deficits but did not improve lifespan in D42-GAL4>TDP-43^wt^ and D42-GAL4>TDP-43 G298S fly models. No improvement in SOD1. Significant reduction in N-acetylglutamine, linked to glutamate excitotoxicity [108].
Rapamycin	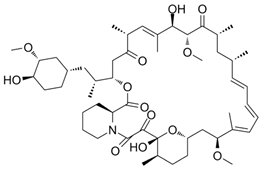	A quantity of 400 µM caused toxicity in control flies; however, it partially improved locomotive defects and lifespan in D42-GAL4^ts^>TBPH flies. Treatment did not improve neuron counts in the thoracic ganglia, but it did reduce the number of neurons with TBPH-positive aggregates [111].
nTRD22	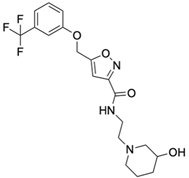	A 50 µM treatment significantly improved motor defects in a D42-GAL4>TDP-43 overexpression fly model. This effect was increased in older (19–20 days) flies vs. younger (5–6 days) flies [114].
rTRD01	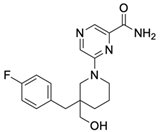	A 20 µM treatment did not improve larvae locomotor deficit in a D42-GAL4>TDP-43 WT model but did so significantly in a D43-GAL4>TDP-43 G298S model (19.3 s vs. 12.3 s larval turning time in untreated vs. treated flies) [113].
NF242	NF242 is a 242 amino acid N-terminal fragment of RGNEF (rho guanine nucleotide exchange factor), capable of binding to TDP-43. RGNEF has been found to co-localize with TDP-43 and other RNA binding proteins implicated in ALS pathology.	Both RGNEF and NF242 co-expression increased lifespan in pan-neuronal and motor neuron-specific TDP-43 fly models. NF242 was able to mitigate a toxic eye phenotype and motor phenotype (GMR, elav, D42 drivers). Immunofluorescence demonstrated that NF242 and TDP-43 WT co-aggregate in both the nucleus and cytosol of neurons [115].

## Data Availability

No new data were created or analyzed in this study. Data sharing is not applicable to this article.

## Abbreviations

ALS, Amyotrophic Lateral Sclerosis; A4V, Ala4Val; C9ORF72, chromosome 9 open reading frame 72; elav, embryonic lethal abnormal vision; FDA, U.S. Food and Drug Administration; FUS, fused in sarcoma; GMR, Glass Multiple Reporter; G85R, Gly85Arg, G93A, Gly93Ala; NIH, National Institutes of Health; nSyb, neuronal Synaptobrevin; ND, neurodegenerative disease; NAM, New Approach Methodology; SOD1, superoxide dismutase 1; TDP-43, TAR-DNA binding protein-43; UAS, upstream activation sequence; TARGET, Temporal and Regional Gene Expression Targeting.
